# Monitoring *Schizosaccharomyces pombe* genome stress by visualizing end-binding protein Ku

**DOI:** 10.1242/bio.054346

**Published:** 2021-02-15

**Authors:** Chance E. Jones, Susan L. Forsburg

**Affiliations:** Program in Molecular & Computational Biology, University of Southern California, Los Angeles, CA 90089, USA

**Keywords:** DNA damage, Ku, Fission yeast, Genome stability, Microscopy

## Abstract

Studies of genome stability have exploited visualization of fluorescently tagged proteins in live cells to characterize DNA damage, checkpoint, and repair responses. In this report, we describe a new tool for fission yeast, a tagged version of the end-binding protein Pku70 which is part of the KU protein complex. We compare Pku70 localization to other markers upon treatment to various genotoxins, and identify a unique pattern of distribution. Pku70 provides a new tool to define and characterize DNA lesions and the repair response.

## Abstract (precis)

**Summary:** The authors describe a fluorescently tagged Ku70 protein to monitor replication stress in live *S. pombe* cells.

## INTRODUCTION

The response to genome stress and DNA repair can be observed in living cells in real time, by monitoring fluorescently-tagged DNA damage response proteins (Lisby et al., 2004; Lukas et al., 2005; Nagy and Soutoglou, 2009; Polo and Jackson, 2011). This has allowed characterization of dynamic processes that respond to damage and preserve genome integrity, including cell cycle, checkpoint, repair, and recovery pathways. In the fission yeast *Schizosaccharomyces pombe*, accumulation of foci of the single-strand DNA binding protein Ssb1 (a subunit of Replication Protein A /RPA), and of the recombination protein Rad52, have been used to characterize intrinsic genome stresses as well as the response to external genotoxins (Meister et al., 2003; Kilkenny et al., 2008; Carneiro et al., 2010; Bass et al., 2012; Sabatinos et al., 2012). These proteins recognize and respond to single strand DNA accumulation, which can result from exonuclease activity, resection, processing of replication forks, and recombination intermediates, or R-loop or D-loop formation (Zeman and Cimprich, 2014; Sabatinos and Forsburg, 2015). Importantly, this has led to identification of distinct patterns of accumulation that can serve as fingerprints for different forms of genome stress (e.g. Sabatinos et al., 2012; Sabatinos and Forsburg, 2015; Ranatunga and Forsburg, 2016).

The fission yeast Pku70 protein is the orthologue of the Ku70 subunit of the conserved heterodimeric Ku complex (Baumann and Cech, 2000). Ku is abundant and binds efficiently to DNA double strand breaks (DSBs) (Fell and Schild-Poulter, 2015; Shibata et al., 2018). Ku is associated with the non-homologous end-joining (NHEJ) mechanism of DNA DSB repair (Mahaney et al., 2009) and protects telomeres (Baumann and Cech, 2000; Ferreira and Cooper, 2001). Additionally, it recognizes ‘one-sided’ DSBs and ends associated with regressed replication forks (Teixeira-Silva et al., 2017; Foster et al., 2011; Langerak et al., 2011).

Ku binding at the ends of DNA inhibits resection and accumulation of single strand DNA that otherwise drives homologous recombination (Shibata et al., 2018). Its activity is coordinated with the Mre11-Rad50-Nbs1 (MRN) protein complex, another early responder to DNA DSBs (Shibata et al., 2018; Syed and Tainer, 2018). MRN is also linked to DNA DSB end binding (Wang et al., 2014) and resection (Shibata et al., 2014) and contributes to DNA damage checkpoint activation (Chahwan et al., 2003; Paull, 2015). The Mre11/Rad32 subunit is able to drive endonucleolytic cleavage of DNA ends that are blocked by covalently bound proteins such as Spo11 or Top2 (Hartsuiker et al., 2009, 2009; Milman et al., 2009; Rothenberg et al., 2009; Garcia et al., 2011; Reginato et al., 2017). To some degree, Ku and MRN act as mutual antagonists; Ku inhibits short-range resection driven by MRN, and MRN removes Ku to facilitate homologous recombination (HR) over NHEJ; and to prevent inappropriate repair of single-end breaks (Langerak et al., 2011; Shao et al., 2012; Myler et al., 2017; Shibata et al., 2018). Interestingly, loss of Ku partly suppresses the sensitivity to DNA damage and replication blocking toxins associated with mutation of MRN (Tomita et al., 2003; Williams et al., 2008; Limbo et al., 2007; Langerak et al., 2011; Teixeira-Silva et al., 2017), which can lead to excessive Exo1-driven resection, but impaired RPA recruitment (Teixeira-Silva et al., 2017).

In this report, we describe the development of a new fluorescent marker for fission yeast, the Pku70 subunit of the Ku protein complex. We constructed a *pku70^+^*-citrine fusion and integrated into the genome in wild-type fission yeast under the endogenous promoter. We examined its behavior and accumulation in treated and untreated wild-type cells in response to different genotoxins. We compared localization of Ku to Rad52, RPA, and Mre11 markers and observe a pattern of foci that is distinct from other proteins. This provides a new tool to characterize responses to different forms of genotoxic stress and a useful addition to the fission yeast tool kit for investigation of the 3-Rs of DNA replication, repair, and recombination.

## RESULTS

### Construction of strains with fluorescently tagged Pku70 and Mre11

**Fig. 1.**
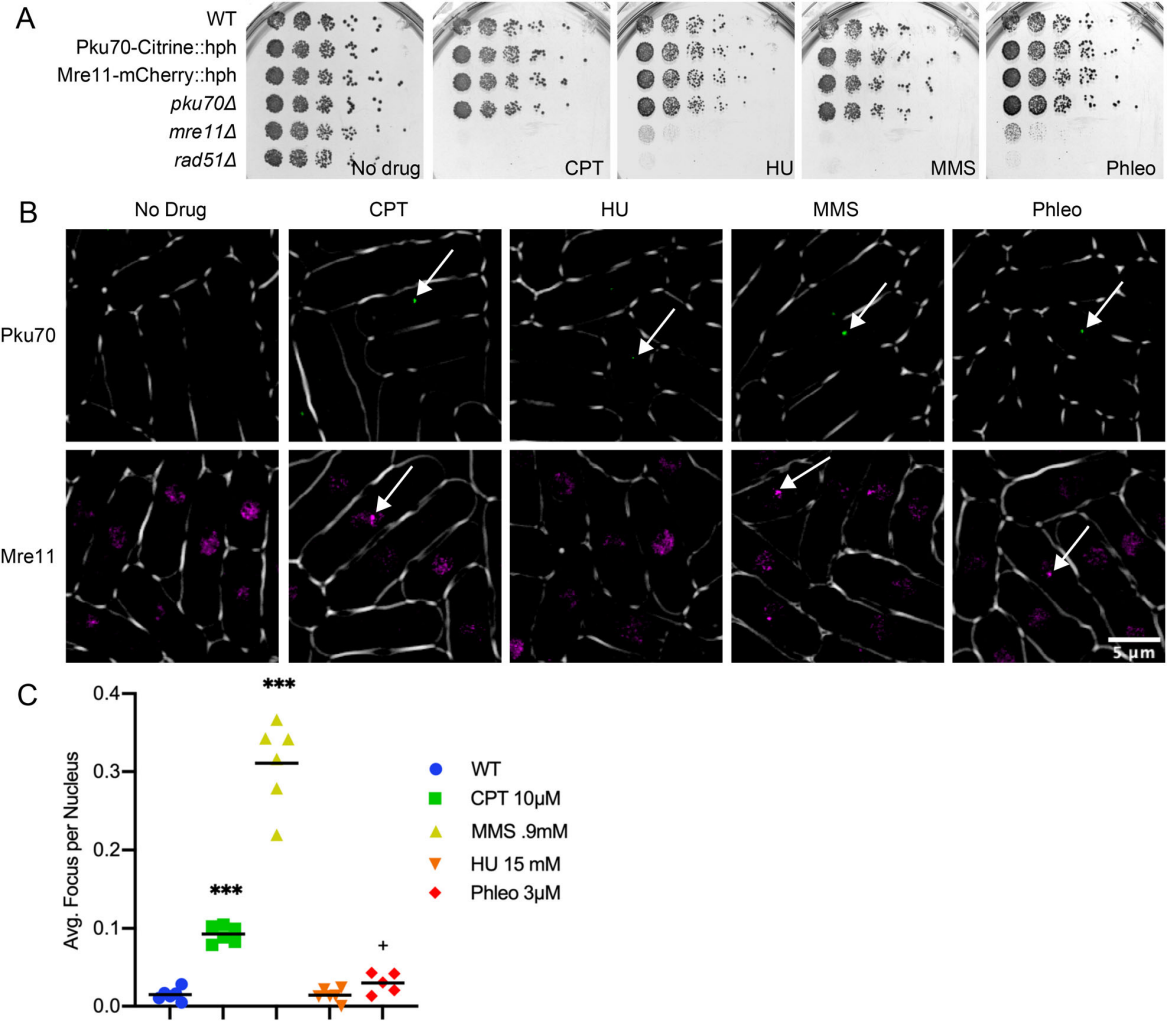
**Construction of fluorescently tagged strains.** (A) Frogging of fluorescently tagged strains on various genotoxic drugs. Drug concentrations are as follows: CPT, 0.0125 mM; HU, 7.5 mM; MMS, 0.012%; Phleo, 1.5 mU. (B) Fluorescently tagged strains Pku70-Citrine::hph and Mre11-mCherry::hph both with and without genotoxic drugs. For clarity Mre11-mCherry is shown in false color as magenta. Arrows show representative foci for Pku70 and generalized areas of increased fluorescence for Mre11. (C) Pku-Citrine foci were counted by hand and significance testing was done using a Mann–Whitney significance test. Six replicates were used per drug tested.

Ku (a heterodimer of Pku70/80) and MRN (Mre11/Rad50/Nbs1) protein complexes are known for high affinity for binding DNA ends (Fell and Schild-Poulter, 2015; Shibata et al., 2018). We tagged Pku70 on its C-terminal end with Citrine fluorescent protein and integrated into the endogenous locus (see the Materials and Methods). Using a similar strategy, we also tagged Mre11 on its C terminal end with mCherry fluorescent protein. The resulting strains were compared to wild-type, *pku70*Δ, *mre11*Δ, and *rad51*Δ for their growth on four typical genotoxic drugs: methyl methanesulfonate (MMS), which creates alkylation damage that inhibits DNA replication fork progression; camptothecin (CPT), which blocks Topoisomerase I cleavage; hydroxyurea (HU), which causes nucleotide starvation and fork pausing; and Phleomycin (phleo), a radio-mimetic that causes single- and DSBs. Both the Mre11-mCherry and Pku70-Citrine tagged strains behaved the same as wild-type under normal growth and genotoxic stress. The *Δpku70* strain also shows no sign of genotoxin sensitivity, as reported previously (Manolis et al., 2001; Sánchez and Russell, 2015) (Fig. 1A). In order to confirm that Pku70-Citrine is active, we performed a plasmid religation assay showing the tagged construct retains NHEJ function. (Fig. S1). We also find that deletion of Pku80 abolishes Pku70-citrine localization, consistent with proper assembly of the Ku heterodimer (Fig. S2).

### Pku70 and Mre11 have increased nuclear signal following genotoxic stress

In normal growth conditions, the tagged strains show a few scattered foci in Pku70-citrine cells and diffuse nuclear fluorescence in Mre11-mCherry cells (Fig. 1B). We examined the distribution of signal in cells treated with MMS, CPT, Phleo, or HU at 32°C after 4 h. There is a significant increase of cells with individual Pku70 nuclear foci in MMS, CPT, and to a lesser extent Phleo. Cells treated with HU did not show any significant difference from wild-type (Fig. 1C). In contrast, the Mre11-mCherry signal showed diffuse pan-nuclear staining in untreated cells (Fig. 1B). Following 4 h of treatment with the four genotoxic drugs, Mre11-mCherry did not show obvious foci. Rather, we observed generalized areas of increased fluorescence over threshold, but these typically were not well-defined discrete puncta as seen with other markers.

### Colocalization of Pku70 and Mre11 with other markers of DNA damage

**Fig. 2.**
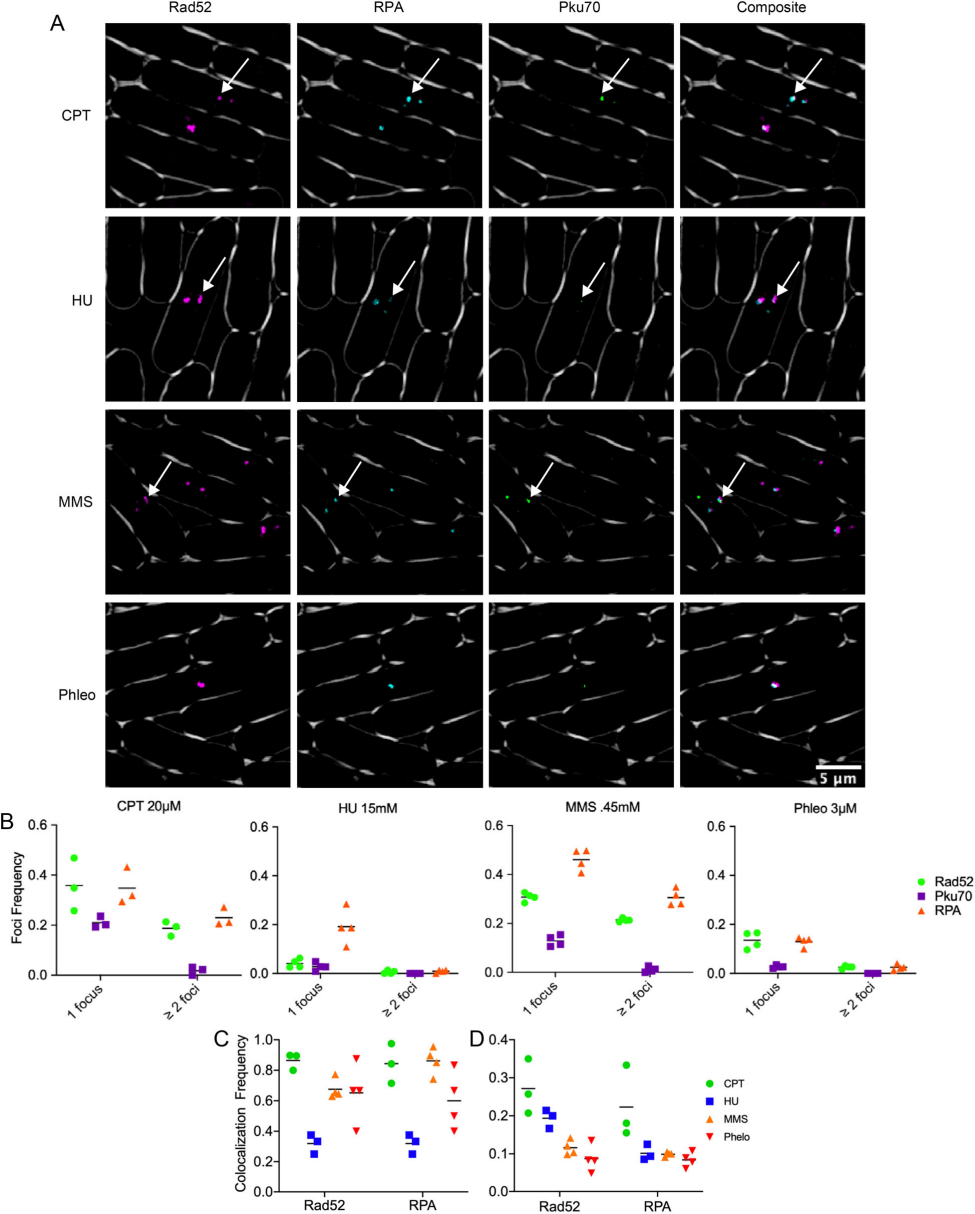
**Pku70-Citrine colocalization with DNA repair proteins.** (A) These images depict colocalization of Pku70-Citrine with previously reported HR repair proteins Rad52-mCherry and RPA-CFP under four commonly used genotoxic drugs. For clarity Rad52-mCherry is shown in false color magenta, Pku70-Citrine is being shown in false color as green. In the composite image overlapping foci appear as white. Arrows show examples of easily visible colocalization. (B) Percent of nuclei with either 1 or ≥2 Pku70-Citrine foci. (C) Percent of Pku70 foci that colocalize with either a Rad52 foci or a RPA foci. (D) Percent of either Rad52 or RPA foci that have a corresponding colocalizing Pku70 foci.

Previous studies of genome instability in fission yeast have imaged the single stranded binding protein Ssb1 (Rad11, RPA) and the homologous recombination protein Rad52 in response to different forms of replication stress (Meister et al., 2003; Kilkenny et al., 2008; Carneiro et al., 2010; Bass et al., 2012; Sabatinos et al., 2012). We examined co-localization using CPT, MMS, HU and Phleo in a strain with Pku70-citrine, Rad52-mCherry, and RPA-CFP. Four hours after drug addition at 32°C, we determined frequency of colocalization among all three tagged proteins. While there was partial overlap, we observed that Pku70 is not completely concordant with the other markers (Fig. 2A).

The number of foci per nucleus was calculated and binned as either 1or ≥2 foci using an automatic foci counter in ImageJ as described in the Materials and Methods (Fig. 2B). We observed that CPT 20 µM contained the highest frequency of Pku70 foci, then MMS, Phleo, and HU. The difference from prior observation likely reflects a somewhat different drug dosage: CPT levels were raised from 10 µM to 20 µM in order to produce an enhanced response and MMS was lowered from 0.9 mM to 0.45 mM to better resolve single foci*.*

Colocalization was determined using the objects-based method in the ImageJ plug-in JACoP (see the Materials and Methods). Fig. 2C shows the proportion of Pku70-Citrine foci that overlap with a thresholded region for Rad52-mCherry or RPA-CFP. For CPT, MMS, and Phleo, these proportions vary from 60–90%. In contrast, the scattered foci in HU showed only about 30% of Ku co-associating with another marker. Fig. 2D shows the proportion of Rad52-mCherry foci that have a colocalizing Pku70-Citrine focus. CPT contained the highest proportion of Rad52 as well as RPA with overlapping Pku70 foci, whereas HU contained the lowest.

**Fig. 3.**
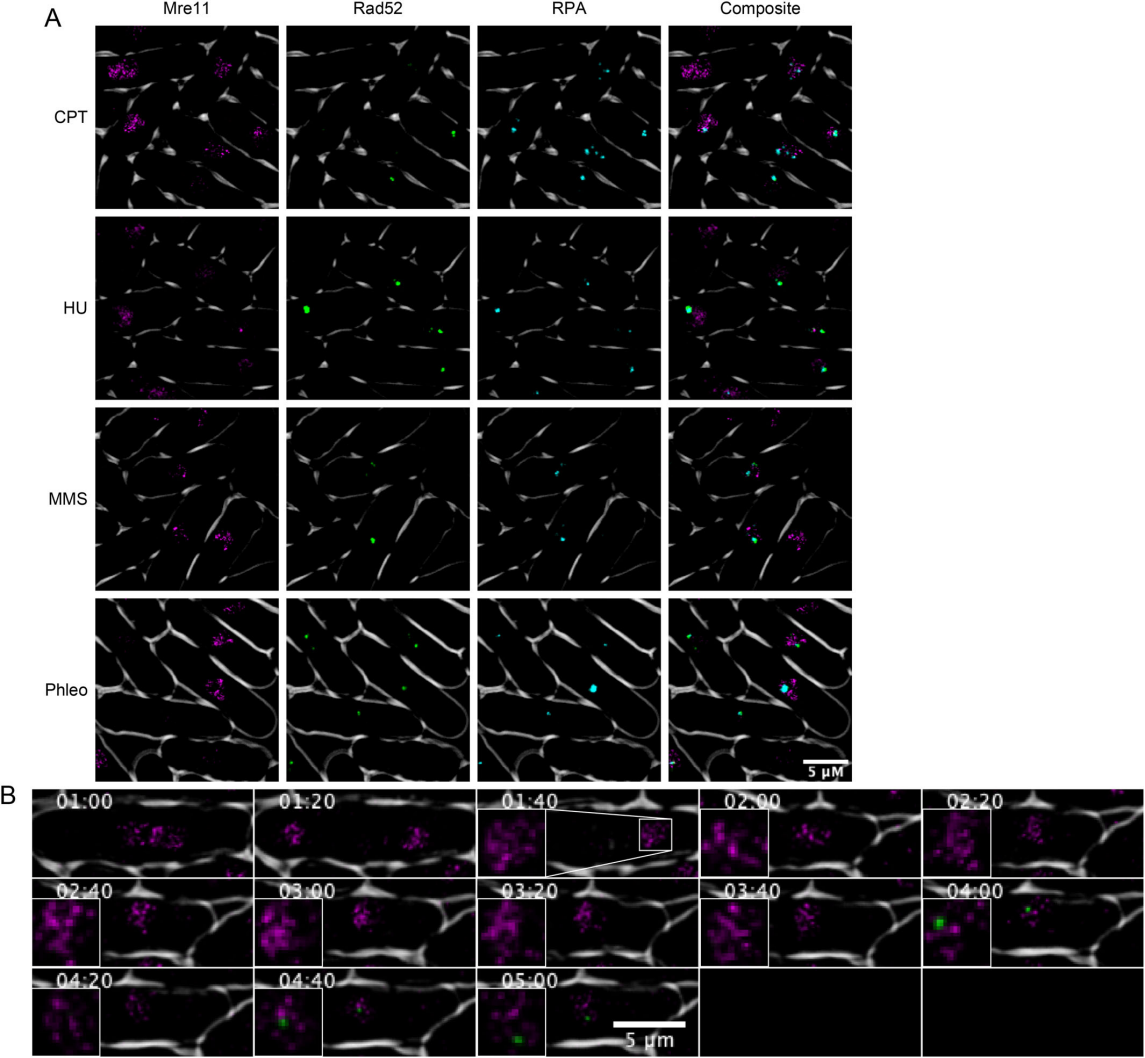
**Colocalization of Mre11-mCherry, Rad52-YFP, RPA-CFP and Pku70-Citrine.** (A) Cells were treated in 0.45 mM MMS for 4 h at 32°C. Mre11-mCherry is shown in false color as magenta and Rad52-YFP is shown in green for clarity. (B) Time-lapse microscopy of Mre11-mCherry and Pku70-Citrine. Cells were treated in 0.45 mM MMS and time-lapses were kept 28°C. Timepoints designate time since drug addition.

We performed a similar study with Mre11-mCherry but could not perform the same quantitation because Mre11-mCherry does not form discrete foci. We observed areas of generally increased fluorescence but never clear puncta as with Pku70, Rad52, or RPA. Observing these cells in three-dimensional reconstruction showed no obvious colocalization between Rad52-YFP/RPA-CFP and Mre11-mCherry in live cell video microscopy, or in static images (Fig. 3A,B; Fig. S3).

### Pku dynamics in S phase specific damage

The genotoxin MMS causes alkylation damage, generating lesions that block DNA polymerase (Lundin et al., 2005). This typically results in replication template switching (Barbour and Xiao 2003; Andersen et al. 2008). Previous work has suggested that Ku is recruited by blocked and regressed replication forks (Teixeira-Silva et al., 2017). Therefore, we investigated the dynamics of Ku response to MMS treatment as a model for disruptions in replication fork progression. We used live cell video microscopy to observe cells containing Rad52-mCherry and Pku70-Citrine over a 5 h period of MMS (0.45 mM) treatment at 28°C. We observe distinct dynamics for Rad52 and Pku70 recruitment during treatment. While absolute timing differs in individual cells, typically a Ku focus appears for a short time and partially co-localizes with Rad52.

**Fig. 4.**
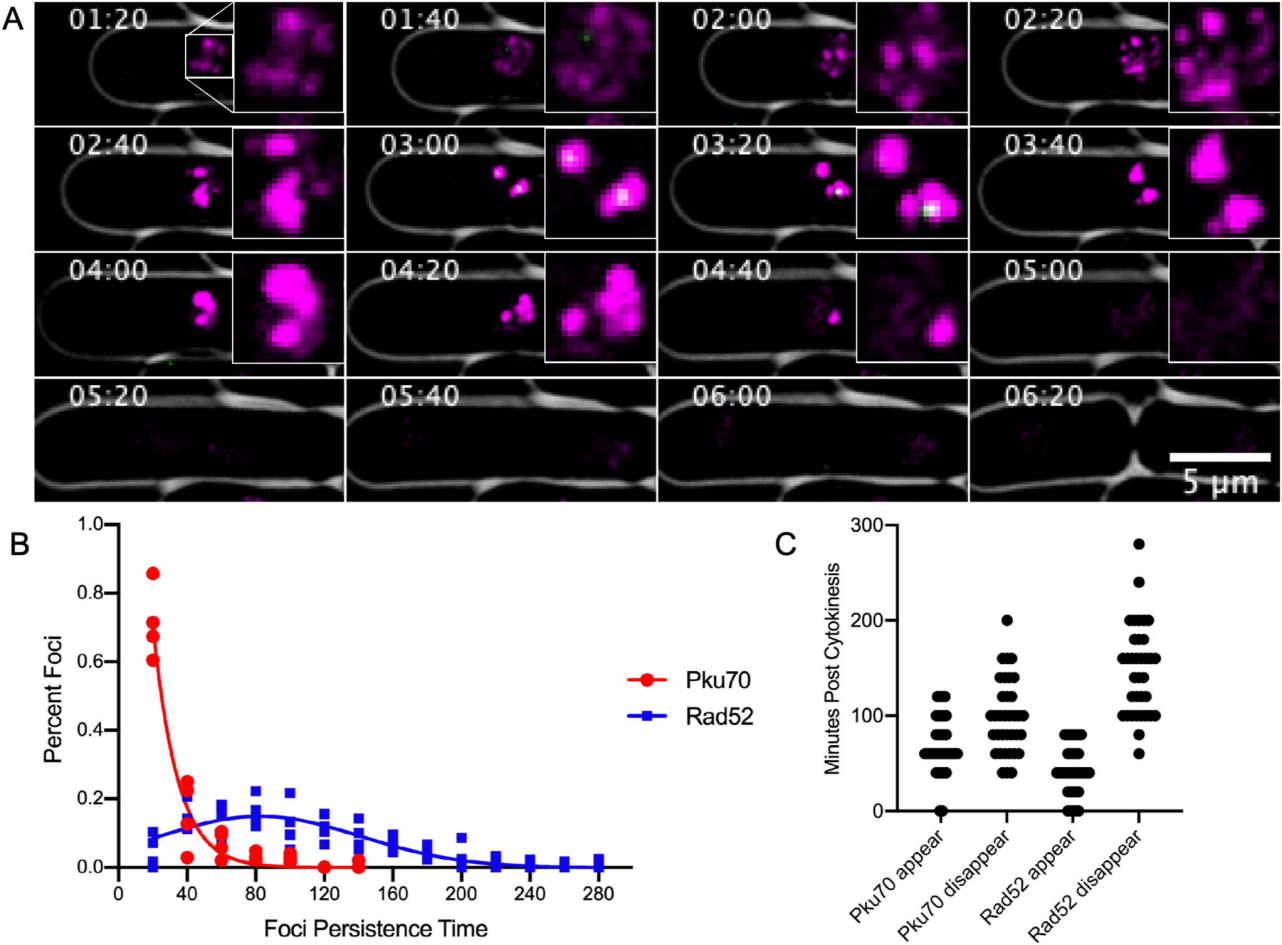
**Pku70 localization in a dynamic timecourse of MMS treatment.** (A) Fluorescent time lapse images of Pku70-Citrine colocalizing with Rad52-mcherry. For clarity Rad52-mCherry is shown in magenta and Pku-Citrine is shown in green. Imaging was started at 80 min post addition of 0.45 mM MMS and cells were imaged at 28°C. Time-course images were taken every 20 min. (B) Persistence time of Rad52-mCherry (*n*=205) and Pku70-Citrine (*n*=195). (C) Appearance and disappearance times for Pku70-Citrine and Rad52-YFP in individual mononucleate cells. T=0 first timepoint after completion of cytokinesis. Horizontal density shows higher quantity of foci appearing or disappearing at that timepoint. (*n*=35 cells).

Fig. 4A shows a representative newborn cell that is likely in mid S phase, 1 h 20 m after drug treatment. The diffuse Rad52-mCherry signal is distributed in smaller foci which then coalesce into two large foci. Pku70-Citrine colocalizes at the center of these large foci for about 20–40 min. The large Rad52-mCherry foci persist for another 60 min and then begin to dissipate. Retention time of Pku-Citrine foci in MMS is ≤20 min with a fraction of cells maintaining it longer between 20 and 40 min. In contrast, Rad52 foci extend over a much longer period of time ranging from 20 all the way up to 160 min (Fig. 4B). Overall Rad52 tends to appear slightly earlier than Pku70 in most cells and disappears much later (Fig. 4C). (Additional time-lapse images found in Figs S4 and S5).

## DISCUSSION

Localization of repair puncta in fission yeast has been a well-established means of observing DNA damage, quantified by counting foci, determining pixel intensity or size of foci, and three-dimensional position in the nucleus (Green et al., 2015). The most frequently used fluorescent tags used in *S. pombe* for observing DNA lesions are the recombination protein Rad52 and single strand DNA binding protein Rad11, a subunit of RPA (Meister et al., 2003; Carneiro et al., 2010; Sabatinos et al., 2012). Studies have shown that in cycling wild-type cells, approximately 10–20% of cells show evidence of single RPA or Rad52 foci, likely due to sporadic S phase damage. The tagged proteins show distinct patterns in response to genotoxic stresses induced by mutations in the replication or repair pathways (Sabatinos et al., 2012; Sabatinos and Forsburg, 2015; Ranatunga and Forsburg, 2016), or in response to exogenous agents such as HU, which causes replication fork stalling (Thelander and Reichard, 1979); MMS, an alkylating agent that generates lesions that block the replication fork (Lundin et al., 2005); camptothecin (CPT), a topoisomerase I inhibitor that leads to S-phase specific double strand breaks (Li and Liu, 2001); and bleo- or phleomycin, radiomimetic drugs that causes single- and DSBs (Povirk, 1996).

The current study seeks to expand the library of tagged proteins, part of our strategy to develop a fingerprint for the response to different forms of genotoxic stress. We investigated fluorescently tagged Mre11 and Pku70 as markers for DNA breaks.

The MRN complex is one of the earliest responders to DSBs (Shibata et al., 2018; Syed and Tainer, 2018) and is essential to drive resection (Wang et al., 2014; Shibata et al., 2018; Langerak et al., 2011; Teixeira-Silva et al., 2017). Our Mre11-mCherry construct showed a diffuse pan-nuclear signal in untreated cells. We did not see obvious focus formation of Mre11-mCherry following treatment with genotoxins. Rather, it maintained a diffuse signal with regions of brightness. In other systems, MRN has been shown to be an immediate responder to DSBs induced by ionizing radiation (Maser et al., 1997). Our failure to see this form of localization may be related to the timing of our analysis, and/or the diffuse distribution of lesions in drug-treated cells, compared to concentrated sites of damage from of ionizing radiation.

Previous whole-cell localization of Pku70 in *S. pombe* was carried out using C terminal epitope-tagged Pku70 and immunofluorescence on fixed cells (Manolis et al., 2001). In unperturbed cells, a diffuse pan-nuclear localization was observed. Association of Ku with DNA ends has been investigated using chromatin immunoprecipitation; in wild-type cells, it is not enriched unless the MRN complex is missing (Langerak et al., 2011; Teixeira-Silva et al., 2017). Visualization of Pku70 in live fission yeast cells has not previously been performed.

We saw few Ku foci in wild-type cells, consistent with the previous immunofluorescence studies. Treatment for 4 h with our panel of genotoxins showed that HU has little to no accumulation of Ku foci. Treatment with CPT causes a modest increase in the fraction of cells with foci at 10 µM and a more dramatic increase at 20 µM. Similarly, phleomycin, a radiomimetic that causes DNA breaks throughout the cell cycle, has a modest but limited increase in foci relative to untreated cells.

We found that the most dramatic increase of cells with Pku70 foci was obtained by treating with MMS at 0.9 mM. MMS is an alkylating agent that results in error-free and error prone base excision repair during S phase, and thus leading to trans lesion synthesis (Memisoglu and Samson, 2000). This induction in MMS is consistent with prior observations suggesting that Ku is recruited to regressed or broken replication forks in order to stabilize the free end (Langerak et al., 2011; Teixeira-Silva et al., 2017). This suggests that even in MRN^+^ cells, there are situations where Ku remains associated with sites of genome stress.

We observed a substantial colocalization between RPA or Rad52 and Ku, in cells treated with MMS, CPT, or Phleo. This result was a surprise as many models suggest Pku should be removed by the time resection and recombination proteins are recruited. One possibility for the S phase specific toxins is that Pku could be binding to reversed forks at repair centers. Previous studies suggest that Pku plays a role at reversed forks in order to maintain genome stability, particularly in cells with defective HR repair such as *brc1Δ* (Sánchez and Russell, 2015; Teixeira-Silva et al., 2017). This may reflect that other mechanisms than exonuclease activity can generate ssDNA, including helicase unwinding and strand invasion.

To address this finding in dynamic conditions, we examined MMS-treated cells as a model for stalled replication forks. Previously, we showed that MMS induces a dramatic increase in RPA and Rad52 foci relative to other genotoxins (Ranatunga and Forsburg, 2016). We observe substantial recruitment of Rad52-mCherry and brief, partial co-localization of Pku70. The Pku70 signal, largely in 1–2 foci, appears after Rad52 and disappears before Rad52 is resolved. Further molecular work will be required to determine what this signal represents.

It is likely that Ku foci will define distinct structures associated with particular forms of replication stress. For example, in a recent study, our lab showed that a mutant *mcm4-dg* with a defect in the MCM helicase accumulates Ku foci (Kim and Forsburg, 2020). This accumulation can be reversed by activation of the Mus81 resolvase. Mus81 is essential for viability in *pku80*Δ *brc1*Δ mutants (Sánchez and Russell, 2015), indicating a collaboration between Ku and Mus81 in response to replication stress. Our Pku70-citrine fusion will be a key reagent in dissecting this and other activities.

## MATERIALS AND METHODS

### Cell growth and physiology

Fission yeast strains are described in Table 1, and were grown as in (Sabatinos et al., 2012).

**Table 1.**
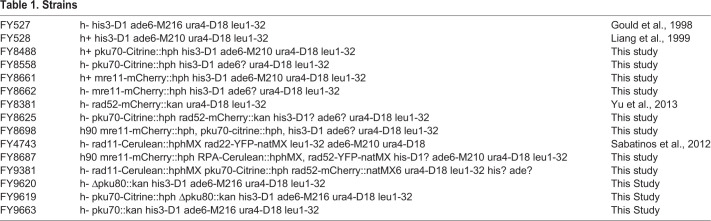
**Strains**

### Construction of tagged strains

All fragments were lengthened using the Expand Long Template PCR System (Roche Diagnostics, Mannheim Germany). Primers were designed using the NCBI Primer design tool and optimized to an annealing temperature of 52–54°C (Ye et al., 2012). Full length fragments were transformed using electroporation and selected using the appropriate marker (Sabatinos and Forsburg, 2010). Upon transformation, instead of plating directly onto selective minimal media, the cells were first plated on YES for 24 h then replica plated onto YES-Hph. Candidate colonies growing on Hph after 4–5 days were then restreaked onto Hph twice and visually screened for nuclear localizing foci.

### Pku-Citrine::Hph

The Pku C-terminal Citrine fragment was formed from five fragments, Pku 5'overhang (FY2710+FY2711), Citrine (FY2561+FY2562), Hph (FY2563+FY2564), Citrine UTR (FY2565+FY2566), and Pku 3′ UTR overhang (FY2712+FY2713). The Citrine and Citrine UTR fragments were lengthened from Addgene plasmid pKT0139 (Sheff and Thorn, 2004). The Hph fragment was lengthened from pFA6a-hphMX6 (Hentges et al., 2005). The 5′ and 3′ UTR overhang fragments were lengthened from phenol:chloroform extracted WT (FY527) DNA (Forsburg and Rhind, 2006). The Citrine, Hph, and Citrine UTR fragments were first lengthened to form a full Citrine::Hph fragment. A single PCR reaction was then done with Pku 5′ overhang, Citrine::Hph, and Pku 3′ UTR overhang fragments forming the full fragment. This fragment was then used for electroporation transformation. All primers used to make these fragments can be found in Table 2.

**Table 2.**
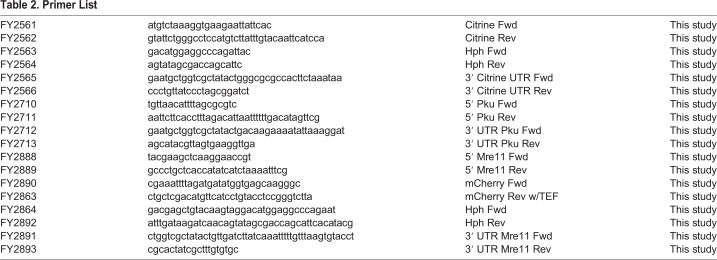
**Primer List**

### Mre11-mCherry::Hph

The Mre11 C-terminal mCherry fragment was formed from four fragments, Mre11 5′ overhang (FY2888+2998), mCherry (FY2890+FY2863), Hph (FY2864+FY2892), Mre11 3′ UTR overhang (FY2891+FY2893). The mCherry fragment was lengthened from extracted DNA, FY8381 (Yu et al., 2013). The Hph fragment was lengthened from the previously formed Citrine::Hph fragment above. The Mre11 5′ and 3′ UTR overhang fragments were lengthened from extracted WT DNA (FY527). The mCherry::Hph fragment was first lengthened. The Mre11 5′ overhang, mCherry::Hph, and Mre11 3′ UTR overhang fragments were then combined in one PCR reaction forming the full fragment. The fragment was then used for electroporation transformation. All primers used to make these fragments can be found in Table 2.

### Plasmid religation assay

Plasmid pAL19 (Barbet et al., 1992) was previously modified by insertion of an *mst1* derivative in the HindIII sites of the multiple cloning sequence, creating pRCP44. pRCP44 was digested for one hour at 37°C with HindIII. The digested fragment containing only the linearized backbone pAL19 was gel-extracted. This ensured that any nonlinearized plasmid was not contaminating the sample. All strains contained the *leu1-32* mutation and were transformed by lithium acetate transformation with either the circularized backbone pAL19 or the linearized backbone pAL19 from the digested/extracted fragments of pRCP44 containing the *S. cerevisiae LEU2*+ gene. Colonies were grown on -Leu plates for 5 days. Colony counts were normalized to wild-type plasmid religation versus circular plasmid transformation rates.

### Live cell imaging

Cells were prepared as in (Green et al., 2015). Medium for all live cell imaging was PMG-HULALA (PMG+Histidine, Uracil, Leucine, Adenine, Lysine, Arginine) (225 mg/l each) (Sabatinos and Forsburg, 2010). Unless specified all drug concentrations used for imaging were as follows, MMS 0.9 mM, HU 15 mM, CPT 20 µM, Phleo 3 µM. Strains in liquid cultures at 32°C were grown to mid-log phase. Cells concentrated by a brief microfuge spin were applied to 2% agarose pads made from PMG+HULA and prepared on glass slides sealed with VaLaP (1/1/1 w/w/w vaseline/lanolin/paraffin). Static images were collected at room temperature 22°C and long term time-lapse images were taken at a constant temperature of 28°C. Images were acquired with a DeltaVision Core (Applied Precision, Issaquah, WA, USA) microscope using a 60× N.A. 1.4 PlanApo objective lens and a 12-bit Photometrics CoolSnap HQII CCD. The system x-y pixel size is 0.109 µm. softWoRx v4.1 (Applied Precision, Issaquah, WA, USA) software was used at acquisition. Excitation illumination was from a Solid-state illuminator, CFP was excited and detected with a 438/24,470/24 filter set (excitation intensity attenuated to 10%) and a 400 ms exposure; YFP was excited and detected with a 513/17,559/38 (excitation intensity attenuated to 32% for Rad52-YFP and 50% for Pku70-Citrine) filter set and a 200 ms exposure. A suitable polychroic mirror was used. Sections of static timepoints were 20 0.20 µm z-sections. Long-term time-lapse videos used 8 z-steps of 0.35 µm. Three-dimensional stacks were deconvolved with manufacturer provided OTFs using a constrained iterative algorithm, images were maximum intensity projected for presentation. Images were contrast adjusted using a histogram stretch with an equivalent scale and gamma for comparability. Brightfield images were also acquired.

### Image processing and analysis

Images were contrast adjusted using an equivalent histogram stretch on all samples. Significance was assessed with Mann–Whitney tests. Long-term time lapse videos were stabilized in ImageJ-Fiji (Schindelin et at. 2012) using the package ‘StackReg’ by Philippe Thevanaz from the Biomedical Imaging Group at the Swiss Federal Institute of Technology Lausanne (Thevenaz et al., 1998). Foci were automatically quantified using a computational algorithm based on uniform threshold per fluorescence channel as described by the light microscopy core facility at Duke University (https://microscopy.duke.edu/guides/count-nuclear-foci-ImageJ). Object based colocalization analysis was performed using the ImageJ plug-in JACoP on the same images used for the focus quantification. However this object based colocalization analysis method still requires observer-based thresholding before analysis. In order to mitigate observer-based thresholding bias, the number of observed objects after thresholding per fluorescence channel was calculated to be within 10 foci of the automatically counted foci during the previous computer-based foci quantification analysis described above.

## Supplementary Material

Supplementary information

## References

[BIO054346C57] Andersen, P. L., Xu, F. and Xiao, W. (2008). Eukaryotic DNA damage tolerance and translesion synthesis through covalent modifications of PCNA. *Cell Research*, 18, 162–173. 10.1038/cr.2007.114 18157158

[BIO054346C1] Barbet, N., Muriel, W. J. and Carr, A. M. (1992). Versatile shuttle vectors and genomic libraries for use with Schizosaccharomyces pombe. *Gene* 114, 59-66. 10.1016/0378-1119(92)90707-V 1587485

[BIO054346C58] Barbour, L. and Xiao, W. (2003). Regulation of alternative replication bypass pathways at stalled replication forks and its effects on genome stability: a yeast model. *Mutation Research/Fundamental and Molecular Mechanisms of Mutagenesis*, 532, 137–155. 10.1016/j.mrfmmm.2003.08.014 14643434

[BIO054346C2] Bass, K. L., Murray, J. M. and O'Connell, M. J. (2012). Brc1-dependent recovery from replication stress. *Journal of Cell Science*, 125, 2753–2764. 10.1242/jcs.103119 22366461 PMC3403237

[BIO054346C3] Baumann, P. and Cech, T. R. (2000). Protection of telomeres by the Ku protein in fission yeast. *Mol. Biol. Cell* 11, 3265-3275. 10.1091/mbc.11.10.3265 11029034 PMC14990

[BIO054346C4] Carneiro, T., Khair, L., Reis, C. C., Borges, V., Moser, B. A., Nakamura, T. M. and Ferreira, M. G. (2010). Telomeres avoid end detection by severing the checkpoint signal transduction pathway. *Nature* 467, 228-232. 10.1038/nature09353 20829797 PMC3196630

[BIO054346C5] Chahwan, C., Nakamura, T. M., Sivakumar, S., Russell, P. and Rhind, N. (2003). The fission yeast Rad32 (Mre11)-Rad50-Nbs1 complex is required for the S-phase DNA damage checkpoint. *Mol. Cell. Biol.* 23, 6564-6573. 10.1128/MCB.23.18.6564-6573.2003 12944482 PMC193710

[BIO054346C6] Fell, V. L. and Schild-Poulter, C. (2015). The Ku heterodimer: function in DNA repair and beyond. *Mutat. Res. Rev. Mutat. Res.* 763, 15-29. 10.1016/j.mrrev.2014.06.002 25795113

[BIO054346C7] Ferreira, M. G. and Cooper, J. P. (2001). The fission yeast Taz1 protein protects chromosomes from Ku-dependent end-to-end fusions. *Mol. Cell* 7, 55-63. 10.1016/S1097-2765(01)00154-X 11172711

[BIO054346C8] Forsburg, S. L. and Rhind, N. (2006). Basic methods for fission yeast. *Yeast* 23, 173-183. 10.1002/yea.1347 16498704 PMC5074380

[BIO054346C9] Foster, S. S., Balestrini, A. and Petrini, J. H. (2011). Functional interplay of the Mre11 nuclease and Ku in the response to replication-associated DNA damage. *Mol. Cell. Biol.* 31, 4379-4389. 10.1128/MCB.05854-11 21876003 PMC3209331

[BIO054346C10] Garcia, V., Phelps, S. E., Gray, S. and Neale, M. J. (2011). Bidirectional resection of DNA double-strand breaks by Mre11 and Exo1. *Nature* 479, 241-244. 10.1038/nature10515 22002605 PMC3214165

[BIO054346C11] Green, M. D., Sabatinos, S. A. and Forsburg, S. L. (2015). Microscopy techniques to examine DNA replication in fission yeast. In *DNA Replication*, pp. 13-41. New York, NY: Humana Press. 10.1007/978-1-4939-2596-4_2 25916703

[BIO054346C12] Gould, K. L., Burns, C. G., Feoktistova, A., Hu, C. P., Pasion, S. G. and Forsburg, S. L. (1998). Fission yeast cdc24+ encodes a novel replication factor required for chromosome integrity. *Genetics* 149, 1221-1233. 9649516 10.1093/genetics/149.3.1221 PMC1460225

[BIO054346C13] Hartsuiker, E., Mizuno, K., Molnar, M., Kohli, J., Ohta, K. and Carr, A. M. (2009). Ctp1CtIP and Rad32Mre11 nuclease activity are required for Rec12Spo11 removal, but Rec12Spo11 removal is dispensable for other MRN-dependent meiotic functions. *Mol. Cell. Biol.* 29, 1671-1681. 10.1128/MCB.01182-08 19139281 PMC2655602

[BIO054346C14] Hentges, P., Van Driessche, B., Tafforeau, L., Vandenhaute, J. and Carr, A. M. (2005). Three novel antibiotic marker cassettes for gene disruption and marker switching in Schizosaccharomyces pombe. *Yeast* 22, 1013-1019. 10.1002/yea.1291 16200533

[BIO054346C15] Kilkenny, M. L., Doré, A. S., Roe, S. M., Nestoras, K., Ho, J. C., Watts, F. Z. and Pearl, L. H. (2008). Structural and functional analysis of the Crb2–BRCT2 domain reveals distinct roles in checkpoint signaling and DNA damage repair. *Genes Dev.* 22, 2034-2047. 10.1101/gad.472808 18676809 PMC2492745

[BIO054346C16] Kim, S. M. and Forsburg, S. L. (2020). Active replication checkpoint drives genome instability in fission yeast mcm4 mutant. *Mol. Cell. Biol.* 40, e00033-20 10.1128/MCB.00033-20 32341083 PMC7324846

[BIO054346C17] Langerak, P., Mejia-Ramirez, E., Limbo, O. and Russell, P. (2011). Release of Ku and MRN from DNA ends by Mre11 nuclease activity and Ctp1 is required for homologous recombination repair of double-strand breaks. *PLoS Genet.* 7, e1002271 10.1371/journal.pgen.1002271 21931565 PMC3169521

[BIO054346C18] Liang, D. T., Hodson, J. A. and Forsburg, S. L. (1999). Reduced dosage of a single fission yeast MCM protein causes genetic instability and S phase delay. *J. Cell Sci.* 112, 559-567. 9914167 10.1242/jcs.112.4.559

[BIO054346C19] Limbo, O., Chahwan, C., Yamada, Y., De Bruin, R. A., Wittenberg, C. and Russell, P. (2007). Ctp1 is a cell-cycle-regulated protein that functions with Mre11 complex to control double-strand break repair by homologous recombination. *Mol. Cell* 28, 134-146. 10.1016/j.molcel.2007.09.009 17936710 PMC2066204

[BIO054346C20] Li, T. K. and Liu, L. F. (2001). Tumor cell death induced by topoisomerase-targeting drugs. *Annu. Rev. Pharmacol. Toxicol.* 41, 53-77. 10.1146/annurev.pharmtox.41.1.53 11264450

[BIO054346C21] Lisby, M., Barlow, J. H., Burgess, R. C. and Rothstein, R. (2004). Choreography of the DNA damage response: spatiotemporal relationships among checkpoint and repair proteins. *Cell* 118, 699-713. 10.1016/j.cell.2004.08.015 15369670

[BIO054346C22] Lukas, C., Bartek, J. and Lukas, J. (2005). Imaging of protein movement induced by chromosomal breakage: tiny ‘local'lesions pose great ‘global'challenges. *Chromosoma* 114, 146-154. 10.1007/s00412-005-0011-y 15988581

[BIO054346C23] Lundin, C., North, M., Erixon, K., Walters, K., Jenssen, D., Goldman, A. S. and Helleday, T. (2005). Methyl methanesulfonate (MMS) produces heat-labile DNA damage but no detectable in vivo DNA double-strand breaks. *Nucleic Acids Res.* 33, 3799-3811. 10.1093/nar/gki681 16009812 PMC1174933

[BIO054346C24] Mahaney, B. L., Meek, K. and Lees-Miller, S. P. (2009). Repair of ionizing radiation-induced DNA double-strand breaks by non-homologous end-joining. *Biochem. J.* 417, 639-650. 10.1042/BJ20080413 19133841 PMC2975036

[BIO054346C25] Manolis, K. G., Nimmo, E. R., Hartsuiker, E., Carr, A. M., Jeggo, P. A. and Allshire, R. C. (2001). Novel functional requirements for non–homologous DNA end joining in Schizosaccharomyces pombe. *EMBO J.* 20, 210-221. 10.1093/emboj/20.1.210 11226171 PMC140209

[BIO054346C26] Maser, R. S., Monsen, K. J., Nelms, B. E. and Petrini, J. H. (1997). hMre11 and hRad50 nuclear foci are induced during the normal cellular response to DNA double-strand breaks. *Mol. Cell. Biol.* 17, 6087-6096. 10.1128/MCB.17.10.6087 9315668 PMC232458

[BIO054346C27] Meister, P., Poidevin, M., Francesconi, S., Tratner, I., Zarzov, P. and Baldacci, G. (2003). Nuclear factories for signalling and repairing DNA double strand breaks in living fission yeast. *Nucleic Acids Res.* 31, 5064-5073. 10.1093/nar/gkg719 12930957 PMC212815

[BIO054346C28] Memisoglu, A. and Samson, L. (2000). Contribution of base excision repair, nucleotide excision repair, and DNA recombination to alkylation resistance of the fission yeast Schizosaccharomyces pombe. *J. Bacteriol.* 182, 2104-2112. 10.1128/JB.182.8.2104-2112.2000 10735851 PMC111257

[BIO054346C29] Milman, N., Higuchi, E. and Smith, G. R. (2009). Meiotic DNA double-strand break repair requires two nucleases, MRN and Ctp1, to produce a single size class of Rec12 (Spo11)-oligonucleotide complexes. *Mol. Cell. Biol.* 29, 5998-6005. 10.1128/MCB.01127-09 19752195 PMC2772569

[BIO054346C30] Myler, L. R., Gallardo, I. F., Soniat, M. M., Deshpande, R. A., Gonzalez, X. B., Kim, Y., Paull, T. T. and Finkelstein, I. J. (2017). Single-molecule imaging reveals how Mre11-Rad50-Nbs1 initiates DNA break repair. *Mol. Cell* 67, 891-898.e4. 10.1016/j.molcel.2017.08.002 28867292 PMC5609712

[BIO054346C31] Nagy, Z. and Soutoglou, E. (2009). DNA repair: easy to visualize, difficult to elucidate. *Trends Cell Biol.* 19, 617-629. 10.1016/j.tcb.2009.08.010 19819145

[BIO054346C32] Paull, T. T. (2015). Mechanisms of ATM activation. *Annu. Rev. Biochem.* 84, 711-738. 10.1146/annurev-biochem-060614-034335 25580527

[BIO054346C33] Polo, S. E. and Jackson, S. P. (2011). Dynamics of DNA damage response proteins at DNA breaks: a focus on protein modifications. *Genes Dev.* 25, 409-433. 10.1101/gad.2021311 21363960 PMC3049283

[BIO054346C34] Povirk, L. F. (1996). DNA damage and mutagenesis by radiomimetic DNA-cleaving agents: bleomycin, neocarzinostatin and other enediynes. *Mutat. Res.* 355, 71-89. 10.1016/0027-5107(96)00023-1 8781578

[BIO054346C35] Ranatunga, N. S. and Forsburg, S. L. (2016). Characterization of a Novel MMS-Sensitive Allele of Schizosaccharomyces pombe mcm4+. *G3* 6, 3049-3063. 10.1534/g3.116.033571 27473316 PMC5068930

[BIO054346C36] Reginato, G., Cannavo, E. and Cejka, P. (2017). Physiological protein blocks direct the Mre11–Rad50–Xrs2 and Sae2 nuclease complex to initiate DNA end resection. *Genes Dev.* 31, 2325-2330. 10.1101/gad.308254.117 29321179 PMC5795779

[BIO054346C37] Rothenberg, M., Kohli, J. and Ludin, K. (2009). Ctp1 and the MRN-complex are required for endonucleolytic Rec12 removal with release of a single class of oligonucleotides in fission yeast. *PLoS Genet.* 5, e1000722 10.1371/journal.pgen.1000722 19911044 PMC2768786

[BIO054346C38] Sabatinos, S. A. and Forsburg, S. L. (2010). Molecular genetics of Schizosaccharomyces pombe. In *Methods in Enzymology*, Vol. 470, pp. 759-795. Academic Press. 20946835 10.1016/S0076-6879(10)70032-X

[BIO054346C39] Sabatinos, S. A. and Forsburg, S. L. (2015). Managing single-stranded DNA during replication stress in fission yeast. *Biomolecules* 5, 2123-2139. 10.3390/biom5032123 26393661 PMC4598791

[BIO054346C40] Sabatinos, S. A., Green, M. D. and Forsburg, S. L. (2012). Continued DNA synthesis in replication checkpoint mutants leads to fork collapse. *Mol. Cell. Biol.* 32, 4986-4997. 10.1128/MCB.01060-12 23045396 PMC3510540

[BIO054346C41] Sánchez, A. and Russell, P. (2015). Ku stabilizes replication forks in the absence of Brc1. *PLoS ONE* 10, e0126598 10.1371/journal.pone.0126598 25965521 PMC4428774

[BIO054346C42] Schindelin, J., Arganda-Carreras, I. and Frise, E., Kaynig, V., Longair, M., Pietzsch, T., Preibisch, S., Rueden, C., Saalfeld, S., Schmid, B. et al. (2012). Fiji: an open-source platform for biological-image analysis. *Nat. Methods* 9, 676-682. 10.1038/nmeth.2019 22743772 PMC3855844

[BIO054346C43] Shao, Z., Davis, A. J., Fattah, K. R., So, S., Sun, J., Lee, K.-J., Harrison, L., Yang, J. and Chen, D. J. (2012). Persistently bound Ku at DNA ends attenuates DNA end resection and homologous recombination. *DNA Repair* 11, 310-316. 10.1016/j.dnarep.2011.12.007 22265216 PMC3297478

[BIO054346C44] Sheff, M. A. and Thorn, K. S. (2004). Optimized cassettes for fluorescent protein tagging in Saccharomyces cerevisiae. *Yeast* 21, 661-670. 10.1002/yea.1130 15197731

[BIO054346C45] Shibata, A., Moiani, D., Arvai, A. S., Perry, J., Harding, S. M., Genois, M.-M., Maity, R., Van Rossum-Fikkert, S., Kertokalio, A., Romoli, F. et al. (2014). DNA double-strand break repair pathway choice is directed by distinct MRE11 nuclease activities. *Mol. Cell* 53, 7-18. 10.1016/j.molcel.2013.11.003 24316220 PMC3909494

[BIO054346C46] Shibata, A., Jeggo, P. and Löbrich, M. (2018). The pendulum of the Ku-Ku clock. *DNA Repair* 71, 164-171. 10.1016/j.dnarep.2018.08.020 30177438

[BIO054346C47] Syed, A. and Tainer, J. A. (2018). The MRE11–RAD50–NBS1 complex conducts the orchestration of damage signaling and outcomes to stress in DNA replication and repair. *Annu. Rev. Biochem.* 87, 263-294. 10.1146/annurev-biochem-062917-012415 29709199 PMC6076887

[BIO054346C48] Teixeira-Silva, A., Saada, A. A., Hardy, J., Iraqui, I., Nocente, M. C., Fréon, K. and Lambert, S. A. (2017). The end-joining factor Ku acts in the end-resection of double strand break-free arrested replication forks. *Nat. Commun.* 8, 1-14. 10.1038/s41467-017-02144-5 29215009 PMC5719404

[BIO054346C49] Thelander, L. and Reichard, P. (1979). Reduction of ribonucleotides. *Annu. Rev. Biochem.* 48, 133-158. 10.1146/annurev.bi.48.070179.001025 382982

[BIO054346C50] Thevenaz, P., Ruttimann, U. E. and Unser, M. (1998). A pyramid approach to subpixel registration based on intensity. *IEEE Trans. Image Process.* 7, 27-41. 10.1109/83.650848 18267377

[BIO054346C51] Tomita, K., Matsuura, A., Caspari, T., Carr, A. M., Akamatsu, Y., Iwasaki, H., Mizuno, K., Ohta, K., Uritani, M., Ushimaru, T. et al. (2003). Competition between the Rad50 complex and the Ku heterodimer reveals a role for Exo1 in processing double-strand breaks but not telomeres. *Mol. Cell. Biol.* 23, 5186-5197. 10.1128/MCB.23.15.5186-5197.2003 12861005 PMC165728

[BIO054346C52] Wang, Q., Goldstein, M., Alexander, P., Wakeman, T. P., Sun, T., Feng, J., Lou, Z., Kastan, M. B. and Wang, X. F. (2014). Rad17 recruits the MRE11–RAD50–NBS1 complex to regulate the cellular response to DNA double–strand breaks. *EMBO J.* 33, 862-877. 10.1002/embj.201386064 24534091 PMC4194111

[BIO054346C53] Williams, R. S., Moncalian, G., Williams, J. S., Yamada, Y., Limbo, O., Shin, D. S., Groocock, L. M., Cahill, D., Hitomi, C., Guenther, G. et al. (2008). Mre11 dimers coordinate DNA end bridging and nuclease processing in double-strand-break repair. *Cell* 135, 97-109. 10.1016/j.cell.2008.08.017 18854158 PMC2681233

[BIO054346C54] Ye, J., Coulouris, G., Zaretskaya, I., Cutcutache, I., Rozen, S. and Madden, T. (2012). Primer-BLAST: A tool to design target-specific primers for polymerase chain reaction. *BMC Bioinformatics* 13, 134 10.1186/1471-2105-13-134 22708584 PMC3412702

[BIO054346C55] Yu, Y., Ren, J. Y., Zhang, J. M., Suo, F., Fang, X. F., Wu, F. and Du, L. L. (2013). A proteome-wide visual screen identifies fission yeast proteins localizing to DNA double-strand breaks. *DNA Repair* 12, 433-443. 10.1016/j.dnarep.2013.04.001 23628481

[BIO054346C56] Zeman, M. K. and Cimprich, K. A. (2014). Causes and consequences of replication stress. *Nat. Cell Biol.* 16, 2-9. 10.1038/ncb2897 24366029 PMC4354890

