# Supplementary material for: Monitoring Schizosaccharomyces pombe genome stress by visualizing end-binding protein Ku
Source: Biol Open. 2021 Feb 15;10(2):bio054346. doi: 10.1242/bio.054346 (PMC7904001; doi:10.1242/bio.054346)
Supplement: Supplementary information [file biolopen-10-054346-s1.pdf]

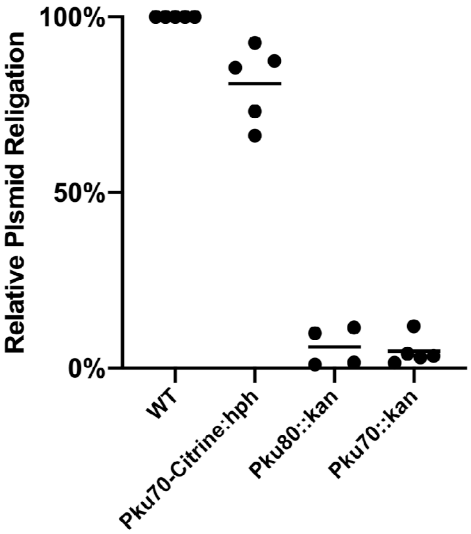

Figure S1. Pku-Citrine is competent for NHEJ repair. All strains contained the leu1-32 mutation and were transformed by lithium acetate transformation with either a circularized plasmid or digested fragment containing the *S. cerivisiae* LEU2+ gene. Colonies were grown on -leu plates for 5 days. Colony counts were normalized to WT plasmid religation vs circular plasmid transformation rates. The middle bar represents the mean of the biological replicates.

Biology Open • Supplementary information

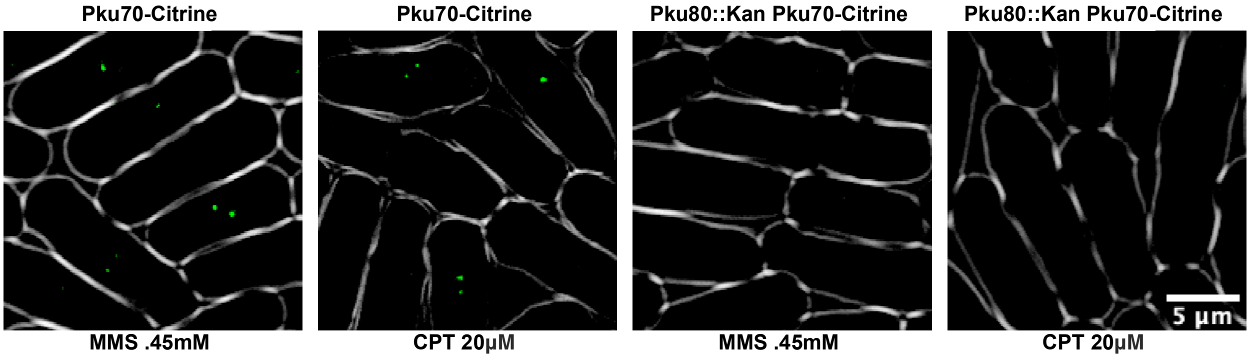

Figure S2. Deletion of Pku80::Kan disrupts Pku70-Citrine localization. Cells were grown in either MMS or CPT for four hours before live cell imaging. Pku70-Citrine is shown in false color as green for clarity.

Biology Open • Supplementary information

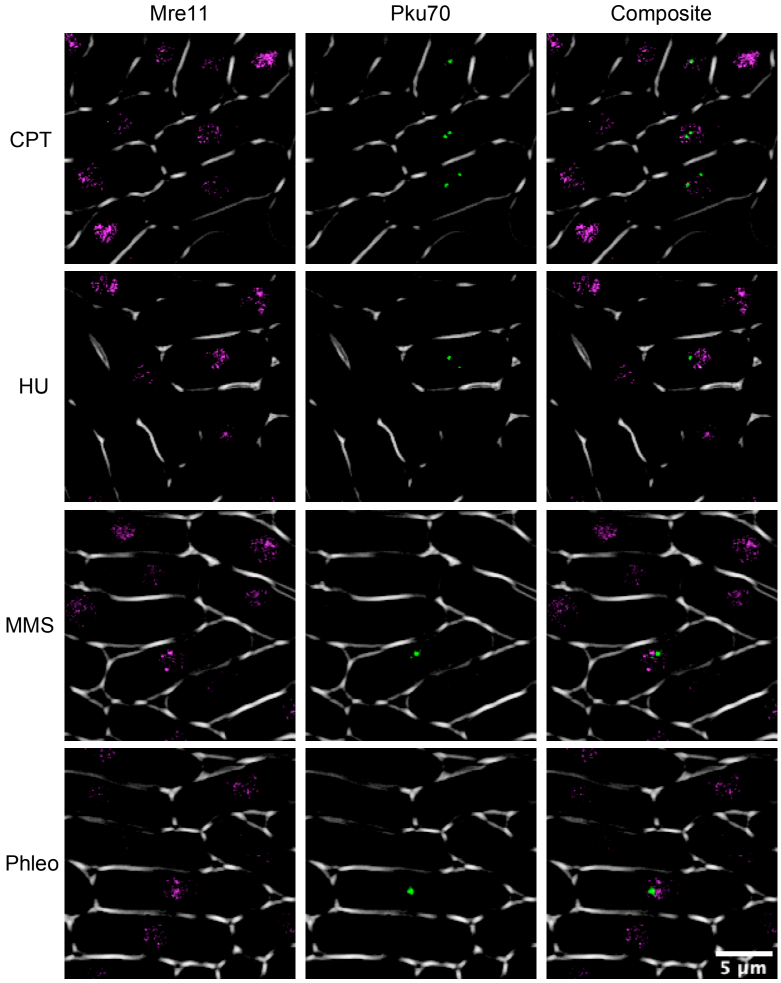

Figure S3. Colocalization of Pku70 and Mre11. Cells were treated in .45mM MMS for 4 hours at 32ºC. Mre11-mCherry is shown in false color as magenta and Pku70 is shown in green for clarity.

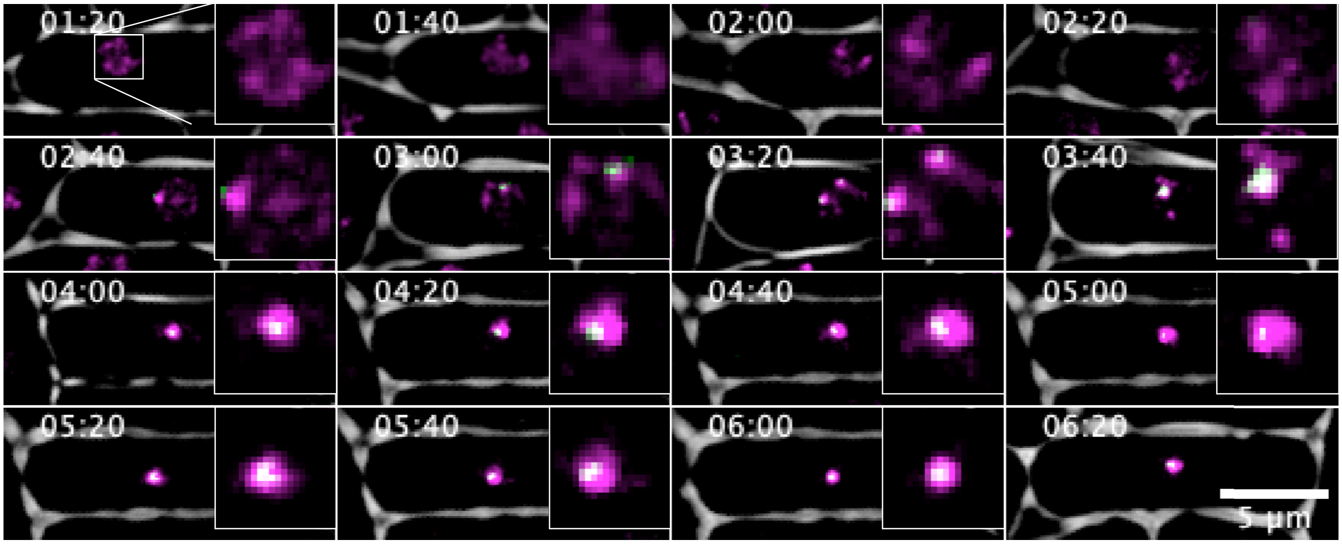

Figure S4. Timelapse of Pku70 and Rad52. Fluorescent time lapse images of Pku70-Citrine colocalizing with Rad52-mcherry. For clarity Rad52-mCherry is shown in magenta as well as Pku-Citrine being shown in green. Imaging was started at 80 minutes post addition of .45mM MMS and cells were grown at 28ºC. Time-course images were taken every 20 min.

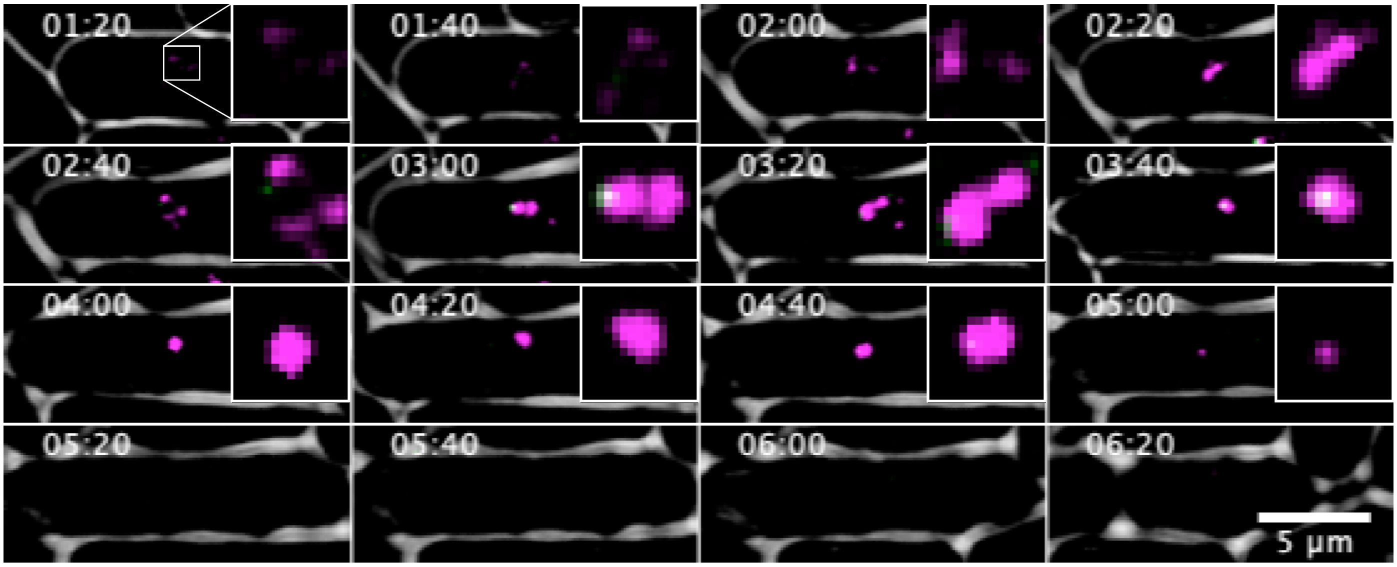

Figure S5. Timelapse of Pku70 and Rad52. Fluorescent time lapse images of Pku70-Citrine colocalizing with Rad52-mcherry. For clarity Rad52-mCherry is shown in magenta as well as Pku-Citrine being shown in green. Imaging was started at 80 minutes post addition of .45mM MMS and cells were grown at 28ºC. Time-course images were taken every 20 min.
